# What Is the Role of Basal Weekly Insulin in Clinical Practice? The State of the Art

**DOI:** 10.3390/biomedicines12040900

**Published:** 2024-04-18

**Authors:** Christiano Argano, Laura Priola, Francesco Manno, Salvatore Corrao

**Affiliations:** 1 Department of Internal Medicine, National Relevance and High Specialization Hospital Trust ARNAS Civico, Di Cristina, Benfratelli, 90127 Palermo, Italy; laura.priola@libero.it (L.P.); francescomanno0@gmail.com (F.M.); s.corrao@tiscali.it (S.C.); 2 Department of Health Promotion Sciences, Maternal and Infant Care, Internal Medicine and Medical Specialties (PROMISE), University of Palermo, 90127 Palermo, Italy

**Keywords:** weekly insulin, icodec, LY3209590 basal insulin Fc (BIF), type 1 diabetes mellitus, type 2 diabetes mellitus

## Abstract

Despite the advent of innovative therapies in the treatment of diabetes, ever-increasing awareness is still directed to the role of insulin since it has continued to be at the centre of diabetes therapy for decades, as a therapeutic integration of innovative agents in type 2 diabetes mellitus (T2DM), as the only replacement therapy in type 1 diabetes mellitus (T1DM) and also in gestational diabetes. In this context, the study of molecules such as weekly basal insulins, both for their technological and pharmacodynamic innovation and their manageability and undoubted benefits in compliance with drug therapy, can only be a turning point in diabetes and for all its phenotypes. This review aims to provide insight into the knowledge of basal weekly insulins and their use in type 1 and 2 diabetes mellitus by examining their safety, efficacy, manageability and increased therapeutic compliance.

## 1. Introduction

Globally, diabetes represents a considerable burden to healthcare systems with an increasing prevalence, primarily due to a rise in obesity [1]. In 2021, the global age-standardized total prevalence was 6.1% (5.8–6.5) [1], resulting in health expenditures of U.S. $966 billion that are expected to rise, reaching more than $1054 billion by 2045 [2]. According to the Global Burden of Diseases, Injuries, and Risk Factors Study (GBD) 2019, diabetes was the eighth cause of death and disability globally [3]. Furthermore, the global burden of diabetes is predicted to increase among elderly patients due to reduced physical activity relating to T2DM mellitus, unhealthy diets, rising incidences of T1DM and the aging of the world population, determining a further surge in the hospitalization of subjects with diabetes and comorbidities, which are essential determinants of diabetes burden in terms of their considerable impact on a patient’s quality of life, health status and outcomes [4,5]. In 2021, approximately 530 million adults worldwide were affected by diabetes [2], and 11.6% of the U.S. population (38.4 million people of all ages) had diabetes. In particular, 14.7% of all U.S. adults (38.1 million adults million adults aged 18 years or older) had diabetes. A total of 35 per 10,000 children and adolescents younger than age 20 years (352,000) had been diagnosed with diabetes. This datum includes 304,000 with type 1 diabetes [1,6]. In Europe, the prevalence of diabetes is 9.2%, and the number of people with diabetes (61 million) will increase to 13% by 2045 [7]. Nowadays, insulin continues to be an agent of ordinary and necessary use in the pharmacological treatment of diabetes, firstly in T1DM and in gestational diabetes, where insulin is the only pharmacological option and the only considerable replacement therapy. Secondly, the treatment of T2DM also takes into account the cardiovascular risk, often as an add-on to other molecules, in order to achieve the pre-established glycemic objectives despite the various pharmacological alternatives.

According to the National Statistics Report of the Centers for Disease Control and Prevention, 5.7% of all U.S. adults (1.7 million adults aged 20 years or older) with diagnosed diabetes use insulin. A total of 12.3% (3.6 million adults aged 20 years or older) of all U.S. adults with diagnosed diabetes started using insulin within a year of their diagnosis [6].

Although the currently available basal insulin formulations are effective and have a reduced hypoglycemic risk compared to past formulations, their therapeutic introduction could be more timely, mainly due to clinical inertia, patient concerns and poor compliance and education by medical personnel [8,9]. Poor adherence to daily dosing is widespread and associated with poor glycemic control [10,11]. Further problems relate to titration based on glycemic compensation and daily needs [12]. In addition, insulin non-adherence was associated with several injection-related factors, such as number of injections, dose calculation and injection technique, interference with daily activities and embarrassment [13,14]. In this sense, to overcome these problems, the research has moved towards developing basal insulin with longer than twenty-four hours of action and a flatter insulin profile [15].

Once-weekly basal insulin administration would reduce clinical inertia, increase treatment adherence and improve patients’ quality of life, provided the risk of hypoglycemia remains low. Comparisons of once-weekly Glucagon-like peptide-1 receptor agonists (GLP-1 RAs) with once-daily GLP-1 RAs come to our aid [16,17,18,19].

Recent studies have showed that once-weekly insulin treatment had glucose-lowering efficacy and a safety profile [20,21]. Given this background, this review explored current knowledge about basal weekly insulins and their use in type 1 and 2 diabetes.

## 2. Methods

An extensive search of SCOPUS, PubMed and CENTRAL was performed using the following string: “(once-weekly insulin) AND (((“Clinical Trial, Phase III” [Publication Type]) OR (“Clinical Trial, Phase II” [Publication Type]) OR “Clinical Trial, Phase IV” [Publication Type]) OR “Randomized Controlled Trial” [Publication Type])” [22,23,24]. The search string retrieved 167 manuscripts. Hand-searching for principal generalist, human nutrition and basic research journals was also carried out. Two authors (L.P. and F.M.) independently reviewed the retrieved articles’ titles, abstracts and full texts to determine their potential inclusion. Any disagreements were resolved via discussion with other authors (S.C and C.A.). Manuscripts regarding the role of weekly insulin in type 1 and 2 diabetes were extracted for this review.

## 3. Basal Weekly Insulin 

Over the last 100 years, insulin therapy has evolved in parallel with advances in biochemistry and biotechnology [25]. Despite numerous milestones over the last 100 years, insulin is still in constant technological development to facilitate compliance [26]. The first insulins were crude preparations from bovine or porcine pancreas. These were associated with side effects such as lipodystrophy and allergic reactions [27,28] and were administered several times a day given their short duration of action. Basal insulin is essential to insulin therapy in T1DM and rapid insulin. In some cases, insulin is necessary to regulate blood sugar levels during fasting at night and after meals in people with T2DM. This is particularly important during episodes of acute glycometabolic decompensation and for individuals who cannot tolerate newer treatments [29]. 

Problems related to therapeutic adherence, quality of life, hypoglycemic risk and secondary disability or the expected compensation often arise [30]. 

Basal insulin is the most common insulin therapy in type 1 and T2DM. It is required for multiple injection therapy in T1DM and can be used as an adjunctive drug in T2DM, along with other drugs usually administered in the decompensation phase. Subsequently, there has been a shift from basal insulin with a duration of action of 5–7 h to once-daily dosing and now to once-weekly dosing with an extended half-life. 

New weekly insulins have been developed, including Fc-fusion proteins of native single-chain insulin and a panel of recombinant native single-chain insulin molecules. In a pre-clinical study [31], these insulins led to a significant decrease in blood glucose levels for five days in db/db mice after a single dose, by more than 50% compared to the controls (*p* < 0.05). Another molecule was PEGylated insulin AB101, which demonstrated activity over seven days in a phase 1 trial. However, its variability in time to onset and drug concentration could be more robust, so it is no longer in development [32]. Another two molecules, the Fc-fusion insulins HM12460A and HM12470, were presented in 2016. Unfortunately, there have been no reports on their progress for several years [33,34,35]. Insumera insulin (PE0139) was analyzed in a randomized controlled phase 2 trial study completed in 2016 (NCT02581657). However, the results have not been published, and it is unknown whether Insumera is still in active development [36]. Currently, two alternative molecules have been further developed: Basal insulin Fc (BIF, LY3209590) and Insulin Icodec.

## 4. Basal Insulin Fc (BIF, LY3209590)

**Figure 1:**
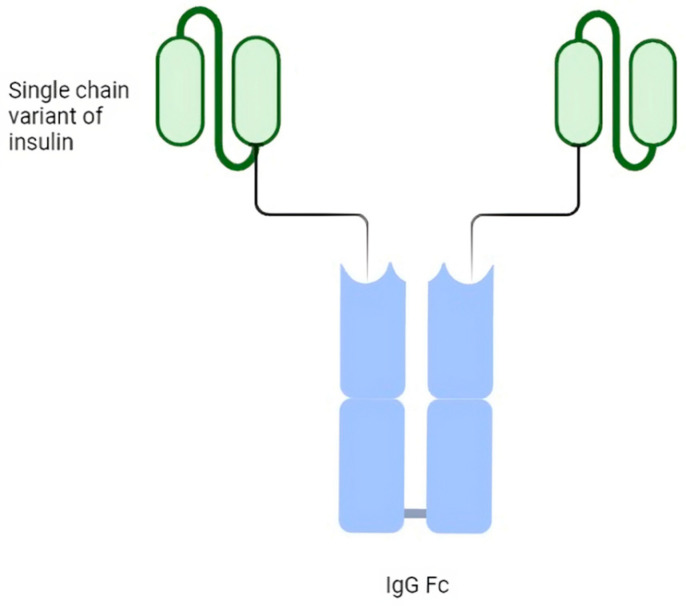
Schematic structure of basal weekly insulin Fc (BIF).

The LY3209590 basal insulin Fc (BIF) is a fusion protein that combines a single-chain insulin variant with a human immunoglobulin G fragment crystallizable domain. It is a selective agonist for insulin receptors and provides full agonism [37]. BIF comprises a human insulin receptor (IR) agonist fused to a human immunoglobulin G2 (IgG2) fragment crystallizable (Fc) domain and has a molecular weight of 64.1 kDa. Each homodimer monomer comprises a single-chain variant of insulin, an interdomain linker and the Fc domain from IgG2. In vitro, the data exhibited a reduced IR-binding affinity, yet with full agonism, selectivity against the insulin-like growth factor-1 receptor and functional properties similar to native human insulin, so it is a selective agonist for insulin receptors and provides full agonism [38] (Figure 1).

**Figure 2:**
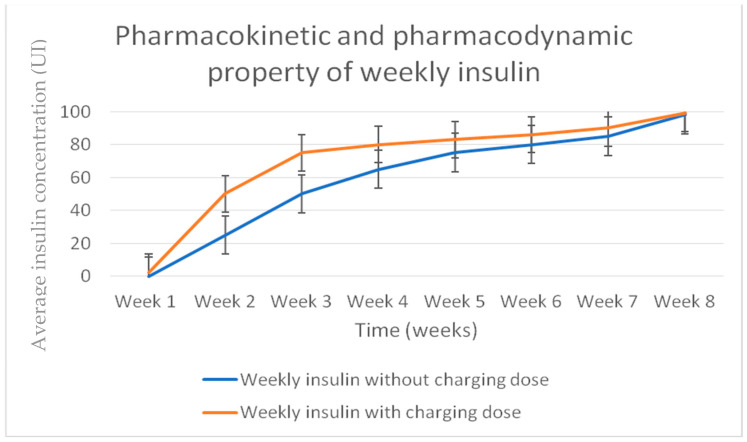
Basal weekly insulin Fc (BIF) pharmacokinetic profile.

It was designed for once-weekly subcutaneous administration in treating patients with T2DM or T1DM. Phase 1 studies indicated that BIF has a low weekly peak-to-trough ratio (1.14, or <15% variation in insulin concentration) and a half-life of 17 days [38] (Figure 2).

## 5. Insulin Icodec

**Figure 3:**
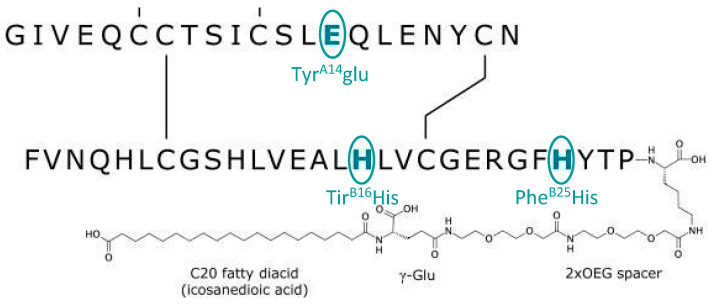
Schematic description and biological properties of basal weekly insulin icodec. The insulin icodec structure shows changes to the human insulin amino acid sequence and chemical modification attached to the lysine in position B29 of insulin.

**Figure 4:**
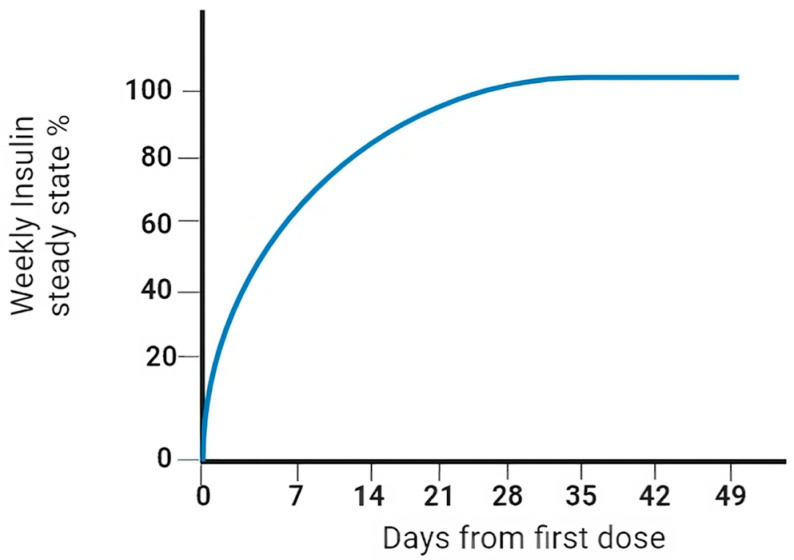
Pharmacokinetic properties (steady state) of weekly insulin.

Icodec insulin is one of two ultra-slow, weekly-acting analogues currently being studied to treat diabetes [39]. In particular, icodec insulin is an acylated analogue (due to the addition of icosanedioic acid, hence the name). It owes its long duration of action to the pharmacodynamic effect that this modification entails, together with the replacement of three amino acids, ensuring a stronger bond with albumin and more remarkable persistence in the bloodstream [40] (Figure 3). By introducing a solid but reversible bond with albumin, icodec guarantees circulating deposition of the drug bound to albumin, which is inactive (through the addition of a side chain containing C20 fatty acid), and three amino acid substitutions (A14E, B16H and B25H), which provides molecular stability with which icodec insulin can activate slowly and steadily, thereby ensuring a prolonged half-life adequate for weekly administration. [40] A clinical pharmacology study demonstrated that icodec has an estimated half-life of 196 h and a uniform hypoglycemic effect throughout the week [40,41] (Figure 4). In vitro cytology studies have demonstrated that icodec activates the same dose-dependent IR-mediated signaling and metabolic responses as endogenous human insulin [41]. Furthermore, the in vitro mitogenic effect of icodec insulin in various human cells was low compared to other types [40,42].

To date, the two most advanced clinical development programs are basal insulin Fc (BIF, LY3209590) (Table 1) and the basal insulin icodec ONWARDS program (Table 2).

## 6. Type 2 Diabetes Mellitus and Weekly Insulin

Even if many new drugs are available now, some patients need to be treated with insulin therapy to achieve personalized glycemic control [51,52]. According to the guidelines, basal insulin needs to be used in a patient with T2DM and severe hyperglycemia (generally blood glucose ≥ 300 mg/dL [≥16.7 mmol/L] or glycated hemoglobin [HbA1c] > 10%) or symptomatic hyperglycemia or if the patient has signs of catabolism (hypertriglyceridemia, weight loss or ketosis) [20]. It is worth outlining that a glycemia level greater than 250 mg/dL [≥13.89 mmol/L] represents a strong predictor of in-hospital mortality in older people hospitalized in internal medicine wards [53]. If a glucagon-like peptide-1 receptor agonist (GLP-1RA) is not suitable, if a more robust approach is needed or if it is a personal preference, insulin therapy is recommended [54,55]. Poor adherence to insulin therapy is a common problem and is responsible for poor outcomes and high healthcare costs. One of the most common causes of reduced adherence is the frequency of injection. This problem could be solved by once-weekly insulin injections, improving patients’ quality of life and leading to better outcomes. This statement is even more pertinent in patients receiving multiple glucose-lowering agents who need injection assistance or are intolerant to other treatments [56]. The rates and reasons for discontinuations vary by study [10,45,46,47,57], but injection frequency is always one important contributing factor. In this sense, the ONWARDS program [39] has been developed to evaluate the safety and efficacy of insulin icodec in T2DM. Six trials are part of it. Five of these trials enrolled T2DM subjects. Going into the specifics of the icodec trial, in the ONWARDS 1 study [48], with head-to-head comparison between icodec and glargine 100 conducted with a large sample of patients in the two groups (492 patients) and with homologous basic characteristics in the two groups, the mean reduction in glycated hemoglobin at 52 weeks and the percentage of TIR (time in range) were evaluated, demonstrating the non-inferiority and superiority of icodec compared to glargine 100. The two groups’ rates of combined clinically significant events or severe hypoglycemia were similar, concluding that once-weekly insulin icodec achieves better glycemic control than once-daily insulin glargine U100.

The ONWARDS 2 [49] study aimed to evaluate the safety and efficacy of once-weekly icodec compared to once-daily insulin degludec in treating T2DM patients already on basal insulin treatment. The study found that once-weekly icodec was better than once-daily degludec in reducing HbA1c levels without causing significant adverse effects.

Similarly, in the ONWARDS 3 [43] study, which focused on insulin-naïve T2DM patients, once-weekly icodec was more effective in reducing HbA1c levels than once-daily degludec after 26 weeks of treatment. The study found no significant difference in secondary outcomes such as weight change and level 2 or 3 hypoglycemic events.

In the clinical trial ONWARDS 4 [58], researchers studied individuals with long-standing T2DM who were on a basal bolus insulin regimen. The study aimed to compare the efficacy of once-weekly insulin icodec to once-daily insulin glargine U100. The results indicated that weekly icodec resulted in better glycemic control with fewer basal insulin injections and lower bolus insulin doses, without increasing the risk of hypoglycemia, in comparison to daily glargine U100.

ONWARDS 5 [59], with attention to technology, is a trial regarding once-weekly insulin codec vs. once-daily basal insulin analogues in people who have T2DM and have not received insulin treatment before with a dosing guide app. Once-weekly icodec resulted in similar improvements in glycemic control compared to once-daily glargine, with fewer basal insulin injections, a lower bolus insulin dose and no increase in hypoglycemic rates compared to glargine U100. Icodec used in conjunction with a dosing guide app demonstrated non-inferiority and superiority versus basal insulin analogues in reducing the estimated mean HbA1c from baseline. A superior time in range was achieved for once-weekly insulin icodec compared with insulin glargine, while the clinically significant or severe hypoglycemia rates were not significantly different between the treatment groups. Weekly BIF was tested in patients with T2DM, achieving a similar efficacy to degludec despite higher fasting glucose targets in the BIF groups for basal insulin Fc. The higher fasting glucose targets and lower glucose variability might have contributed to its lower BIF hypoglycemia rates than degludec [51]. After 26 weeks of treatment once weekly, BIF achieved excellent glycemic control, similar to degludec, with no concerning hypoglycemia in subjects with T2DM [44]. A very recent systematic review [50] demonstrated superior glycometabolic compensation was achieved in patients with T2DM with icodec insulin compared to once-weekly Fc insulin, with no clinically significant differences in major hypoglycemic events.

## 7. Type 1 Diabetes Mellitus and Weekly Insulin

Basal insulin treatment is indispensable for patients with T1DM since it is a replacement therapy. Once-weekly insulin use is more complex in T1DM than in T2DM, but adherence can significantly improve, especially in people prone to missing doses, like teenagers, with better stability and lower episodes of diabetic ketoacidosis. [56,60] Once-weekly BIF demonstrated non-inferior glycemic control to once-daily degludec and no difference in hypoglycemia or other safety findings in patients with T1DM [61].

In ONWARDS 6 [62], the only phase 3 trial regarding the use of once-weekly insulin icodec vs. once-daily insulin degludec in combination with insulin aspart in people with T1DM, insulin icodec was non-inferior to insulin degludec in terms of HbA1c reduction, but severe hypoglycemia episodes occurred in the insulin icodec group.

Nevertheless, patients with diabetes have a positive attitude toward once-weekly injections [26], and a lower frequency of injections is a valuable attribute for injectable therapies [63].

## 8. Discussion 

From a therapeutic point of view, considering the burden of the pathology, insulin continues to be an agent of ordinary and necessary use in the pharmacological treatment of diabetes, especially in T1DM and despite the various pharmacological alternatives for the treatment of T2DM [GLP1-RA, sodium-glucose co-transporter-2 inhibitors (SGLT2i), Dipeptidyl peptidase 4 (DPP4)], often as an add-on to other molecules, in order to achieve the pre-established glycemic objectives [11,57,60]. Although glucose regulation often becomes inadequate with these options as the disease progresses, there is some degree of “clinical inertia” due to the complexity and fear of insulin therapy, both from the perspectives of healthcare providers and people with diabetes. Nevertheless, there are cases of insulinopenic phenotypes where insulin becomes of fundamental use; think of patients with diabetes secondary to pancreaticoduodenectomy, patients with LADA and low c-peptide levels, patients with severe sarcopenia or with side effects and contraindications to GLP1RA or SGLT2i. The challenge in achieving reasonable glycemic control with insulin therapy can be attributed to the complexity of matching the dose and timing of daily insulin injections to the actual physiological requirements. 

In this sense, real-world data from an extensive U.S. electronic medical records database, including 6597 subjects, suggested that among patients with T2DM who initiated basal insulin after oral antidiabetic drugs, the likelihood of reaching glycemic control diminished over time and remained low from 12 months onwards [64]. Another real-world observational study showed that the median time to treatment intensification in patients with elevated HbA1c following basal insulin initiation was 4.3 years [65]. 

The difficulty of integrating insulin use into daily lifestyle due to regimen complexity, reducing the frequency of daily injections and medical affordability are the most common reasons for basal insulin discontinuation [66].

For patients with T1DM, there are currently no therapeutic options available other than insulin therapy using multiple daily insulin injections or micro-infusion pumps. However, despite the advent of innovative therapies for the treatment of patients with T2DM, in many cases, as expressed above, insulin remains a valid option. In this regard, it is delaying the start of insulin therapy in T2DM that results in poor glycometabolic control. Long-acting basal insulin that can exert a hypoglycemic effect in an effective, safe and long-lasting way with just one injection per week should reduce the treatment burden, ensuring better compliance and glycemic control. Clearly, for once-weekly basal insulin to be clinically relevant, it must be comparable to or superior to conventional once-daily insulin treatment in the absence of an increased risk of hypoglycemic events. It remains crucial that both healthcare personnel and patients must learn to switch from conventional insulin therapy, titrate weekly insulin and manage concomitant preprandial insulin if as a basal bolus. At the start of the therapeutic switch, patients switching from once-daily to once-weekly basal insulin may be predisposed to worse initial glycometabolic compensation before reaching a new steady state. However, this can be addressed with a higher initial loading dose and subsequent titration to lower doses, as recently shown with insulin icodec and Fc [38,42,61]. Consistent insulin titration for new users is critical to achieving timely glycemic control [67] and well evidenced, but straightforward titration regimens will be essential to provide confidence in using once-weekly insulins. 

The molecular modifications introduced into insulin icodec and insulin Fc provide novel basal insulin with biological and pharmacokinetic/pharmacodynamic properties suitable for once-weekly dosing. Weekly analogues promise a better quality of life and better therapeutic adherence, reducing the number of injections required from patients.

A clinical phase 3 trial in people with type 1 and 2 diabetes showed that insulin icodec was well tolerated and had pharmacokinetic/pharmacodynamic properties suited for once-weekly dosing. The same is valid for the phase 2 clinical trial of insulin Fc. These once-weekly preparations have demonstrated similar glycemic control to long-acting once-daily insulin analogues, with their hypoglycemic episode rates similar to those of the usual basal insulin preparations. In this sense, a recent meta-analysis showed that insulin icodec was associated with significantly decreased HbA1C, an increased time with glucose in range and similar hypoglycemic and severe adverse effects compared with long-acting insulin in people with T2DM [68]. The possibility of employing basal weekly insulin, beyond its proven safety and efficacy, makes the long duration of action and the reduced need for daily injections noteworthy, as shown by Bajaj and colleagues [69]. All this is undoubtedly associated with better compliance, effectiveness, safety and, consequently, quality of life, which are always to be considered in the management of patients during follow-up. Insulin is a growth factor, and the possibility of reducing the daily dosage per kilogram could, in the long term, reduce the risks linked to its mitotic effect on cells and anabolic effects on tissues, reducing the activation of the insulin receptor and the post-receptor pathways, which may have a stronger mitogenic potency on cancer cells [70]. This argument is a future “open question” for scientific research.

We can, however, consider the use of weekly basal insulin in other rare forms of diabetes that are independent of T1DM or 2 T2DM and which, over time, will be of increasingly more significant scientific interest since there is no solid evidence in place in the literature on the subject. These forms include T2DM patients in the decompensation phase due to steroid therapy, purely “meta steroid” diabetes or patients with LADA. Further application may occur in forms of diabetes secondary to endocrine and exocrine deficiency of the pancreas, such as in chronic pancreatitis or in patients undergoing major pancreatic surgery or severe sarcopenia. Another example, indeed more frequent from an epidemiological point of view, is gestational diabetes, which could be a valid, safe and effective option. Since, a bit like in T1DM, insulin therapy is practically the only valid weapon in gestational diabetes, another open issue remains for gestational diabetes, in which it could be a valid, safe and effective option, reducing the frequency of daily injections; more solid and future clinical data are needed in this regard. These clinical settings represent “open issues” for which there is no current literature but will undoubtedly be of particular scientific interest later on.

## 9. Conclusions

In conclusion, basal weekly long-acting insulin shows similar and better glycemic efficacy than daily basal insulin in T1DM and T2DM due to its association with less hypoglycemia, a reduction in the number of injections and its proven effectiveness. In particular, nowadays, icodec insulin is a candidate to become the primary basal weekly insulin, increasing patient compliance because of its tolerability and encouraging safety results related to hypoglycemia [71]. Consequently, once-weekly insulin could lower the polypharmacy burden among patients with T2DM, primarily comorbid and elderly [4,72], and at the same time, favor their acceptance of insulin therapy.

Although many questions remain unanswered, the future of once-weekly insulin preparations appears bright, and the data regarding some of the clinical issues are encouraging.

## Figures and Tables

**Table 1:** Synthesis of randomized trials regarding insulin Fc (phase 2 trials).

	Insulin Fc vs. Degludec in DMt2 Patients Previously Treated with Basal Insulin [40]	Insulin Fc vs. Degludec in DMt2 Patients Insulino-Naïve [43]	Insulin Fc vs. Degludec in DMt1 Patients [44]
**Study design**	- Multicenter (44 sites) - Randomized, 1:1, open-label - Phase 2 trial - Non-inferiority study vs. basal insulin for efficacy and safety - Basal insulin and up to three oral antidiabetic medicines	- Multicenter (61 sites) - Randomized, 1:1, open-label - Phase 2 trial - Non-inferiority study vs. degludec in DMt2 insulin-naïve patients - Insulin-naïve DMt2 patients previously treated with metformin alone or in combination with dipeptidyl peptidase 4 and/or sodium-glucose cotransporter 2 for at least 3 months prior to screening	- Multicenter (49 sites) - Randomized, 1:1, open-label - Phase 2 trial - Non-inferiority study vs. Degludec in DMt2 insulin-naive patients - Patients with T1D treated with multiple daily basal injections of glargine (U-100 or U-300), detemir, degludec (U-100 or U-200) as basal insulin and as boluses of insulin lispro, aspart, FiAsp or glulisine
**Period**	November 2018–February 2020	5 March 2021–19 July 2023	6 June 2020–22 January 2021
**Endpoint I**	HbA1c reduction at 32 weeks	HbA1c reduction at 26 weeks	HbA1c reduction at 26 weeks
**Endpoint II**	- ΔFPG vs. baseline - Average insulin dose: at weeks 50–52 and 76–78 - Δweight vs. baseline - No. level 2, 3 and combined hypoglycemia vs. baseline - ΔTIR 70–180 mg/dL between groups at weeks 48–52	- Δweight vs. baseline - ΔFPG from baseline to week 26 - No. level 2, 3 and combined hypoglycemia vs. baseline	- Δ percent time in range (TIR) (70–180 mg/dL) on continuous glucose monitoring (CGM) fasting glucose (FG) level - Rate of hypoglycemia
**Titration protocol**	The loading and initial weekly doses were based on their previous daily basal insulin dose and their glycemic control according to baseline HbA1c (using a threshold of 8.5. BIF dosing in the Phase 2 program used mg increments and not insulin international units (IU))	Initial dose 10 IU/day (70 IU/week for icodec) Weekly titration on average FPG of the last 3 days Target: FPG 80–130 mg/dL	Titration was based on mean fasting blood glucose levels using CGM measurements on at least 3 days of the week using a paper-based algorithm. BIF was titrated weekly for weeks 1–12 and then every 4 weeks until the end of the treatment period
**Numbers of patients**	- Enrolled (at least 1 dose): 399 - Completed trials: 351	- Enrolled (at least 1 dose): 278 - Completed trials: 241	- Enrolled (at least 1 dose): 238 - Completed trials: 190
**Population**	- DT2 (average duration 15 years) - Average age 60.2 years - 51% female - Average BMI 30 kg/m^2^ (<32.2 kg/m^2^) - Baseline HbA1c 8.1% (7–11%) - Mean daily basal insulin dose at randomization (39 IU)	- DT2 (average duration 10 years) - Average age 58 years - 45% female - Average BMI 30 kg/m^2^ (<32.2 kg/m^2^) - Baseline HbA1c 8% (7–9.5%) - Mean daily basal insulin dose at randomization (39 IU)	- DT1 (average duration 22 years) - Average age 46 years - 38% female - Average BMI 27.5 kg/m^2^ (<32.2 kg/m^2^) - Baseline HbA1c 7.5% (7–9.5%)
**Results**	- HbA1c reduction −0.1% (CI Δ 0.4–0.03%), *p* < 0.001 - Increase in patients at target (HbA1c < 7%) without significant hypoglycemia: +10% - ΔFPG not significant - Average insulin dose at 52 weeks: 31 IU/day icodec vs. 32 IU/day glargine; on average 0.35 IU/kg/day - Δweight not significant (+2 kg both groups) - TIR increase +4.3% (CI 1.9–6.6%) *p* < 0.001	- At week 26, icodec demonstrated non-inferiority and superiority to degludec in reducing mean HbA1c from baseline. Rates of clinically significant hypoglycemia were not significantly different between treatment groups at week 26.	- At week 26, a non-inferior reduction in HbA1c from baseline was observed compared to patients treated with degludec, with a statistically significant difference of 0.17% (*p* = 0.07) - Time in range (TIR) was similar for patients in the BIF (56.1%) and degludec (58.9%; *p* = 0.112) groups at week 26
**Hypoglycemic events**	The event rates of all documented hypoglycemia were about 25% lower in the Fc groups, and those for nocturnal hypoglycemia were at least 33% lower from baseline to week 32 compared with insulin degludec	The rate of severe hypoglycemic events was not significant between treatment groups (*p* 0.64)	Hypoglycemia occurrence over 24 h was similar for BIF and degludec for level 1 (*p* = 0.960) or level 2 (*p* = 0.517) hypoglycemia during treatment. The occurrence of serious adverse events was similar between the BIF and degludec groups.
**Adverse events**	Mostly mild/moderate events and not associated with treatment Deaths: 3 (2%) in degludec, 1 (1%) in glargine No reactions at the injection site or critical issues related to medication errors described	Mostly mild/moderate events and not associated with treatment: Fc 5.6% (n = 143) Degludec 3% (n = 135) Deaths: 2 (1%) in Fc, 3 (1.5%) in degludec	Mostly mild/moderate events and not associated with treatment. The occurrence of serious adverse events was similar between the BIF and degludec groups.

**Table 2:** Synthesis of randomized trials regarding icodec: the ONWARDS program.

	ONWARDS 1 Icodec vs. Glargine U100 in DT2 Insulino-Naïve [45]	ONWARDS 2 Icodec vs. Degludec U100 in Basal Bolus [46]	ONWARDS 3 Icodec vs. Degludec in DT2 Insulino-Naïve [47]	ONWARDS 4 Icodec vs. Glargine U100 in DT2 in Basal Bolus [48]	ONWARDS 5 Icodec vs. Once-Daily Insulin in DT2 Insulino-Naïve with Dosing Guide App [49]	ONWARDS 6 Icodec vs. Degludec in T1D [50]
**Study design**	- Multicenter (147 sites) - Randomized, 1:1, open-label - Non-inferiority study vs. glargine U100 for efficacy and safety - Basal insulin in addition to any hypoglycemic agent, except S.U. and glinides	- Multicenter (71 sites) - Randomized, 1:1, open-label - Non-inferiority study vs. degludec for efficacy and safety - Basal insulin in insulin-naïve patients, in addition to any hypoglycemic agent, including S.U. and glinides; double-blinded CGM	- Multicenter (92 sites) - Randomized 1:1, double blind - Non-inferiority study vs. degludec for efficacy and safety - Basal insulin in addition to any hypoglycemic agent, including S.U. and glinides	- Multicenter (80 sites) - Randomized, 1:1, open-label - Non-inferiority study vs. glargine U100 for efficacy and safety in patients previously in basal bolus treatment - Basal insulin in addition to any hypoglycemic agent, except S.U. and glinides	- Multicenter (176 sites) - Randomized, 1:1, open-label, parallel group with real-world elements - Non-inferiority study versus once-daily basal insulin analogues (O.D. analogues) dosed per standard practice using a dose-checking app	- Multicenter (99 sites) - Randomized, 1:1, open label - Non-inferiority study vs. degludec for efficacy and safety - Basal insulin in addition to insulin aspart for active group and control group
**Period**	November 2020–May 2023	5 March 2021–19 July 2023	March 2021–June 2022	March 2021–October 2021	1 March 2021–12 August 2022	30 April 2021–15 October 2021
**Endpoint I**	HbA1c reduction at 52 weeks	HbA1c reduction at 26 weeks	HbA1c reduction at 26 weeks	HbA1c reduction at 26 weeks	HbA1c reduction at 52 weeks	HbA1c reduction at 26 weeks
**Endpoint II**	- ΔFPG vs. baseline - Average insulin dose: at weeks 50–52 and 76–78 - Δweight vs. baseline - No. level 2, 3 and combined hypoglycemia vs. baseline - ΔTIR 70–180 mg/dL between groups at weeks 48–52	- Δweight vs. baseline - ΔFPG from baseline to week 26 - No. level 2, 3 and combined hypoglycemia vs. baseline	- ΔFPG vs. baseline - Average insulin dose: at weeks 24–26 - Δweight vs. baseline - No. Level 2, 3 and combined hypoglycemia vs. baseline	- ΔFPG from baseline to week 26 - Δweight vs. baseline - No. level 2, 3 and combined hypoglycemia vs. baseline	- Time from baseline to treatment discontinuation or intensification - No. level 2, 3 and combined hypoglycemia vs. baseline	- HbA1c from baseline to week 52 - ΔFPG from baseline to week 26 - Percentage of time in range (TIR; 3.9–10.0 mmol/L [70–180 mg/dL]) during weeks 22–26 - Δweight vs. baseline - Average insulin dose: at weeks 24–26 and at weeks 50–52 - No. level 2, 3 and combined hypoglycemia vs. baseline
**Titration protocol**	Initial dose of 10 IU/day (70 IU/week for icodec) Weekly titration on average FPG of the last 3 days Target: FPG 80–130 mg/dL	Initial dose of 10 IU/day (70 IU/week for icodec) Weekly titration on average FPG of the last 3 days Target: FPG 80–130 mg/dL	Initial dose of 10 IU/day (70 IU/week for icodec) Weekly titration on average FPG of the last 3 days Target: FPG 80–130 mg/dL Increments of 3 IU/day (20 IU/week for icodec)	Initial dose of 10 IU/day (70 IU/week for icodec) Weekly titration on average FPG of the last 3 days Target: FPG 80–130 mg/dL	Icodec titrated with a dosing guide app (icodec with app)	Initial dose of 10 IU/day (70 IU/week for icodec) Weekly titration on average FPG of the last 3 days Target: FPG 80–130 mg/dL Increments of 3 IU/day (20 IU/week for icodec)
**Numbers of patients**	- Enrolled (at least 1 dose): 984 - Completed trials: 954	- Enrolled (at least 1 dose): 526 - Completed trials: 510	- Enrolled (at least 1 dose): 588 - Completed trials: 564	- Enrolled (at least 1 dose): 582 - Completed trials: 582	- Enrolled (at least 1 dose): 1085 - Completed trials: 1008	- Enrolled (at least 1 dose): 582 - Completed trials: 540
**Population**	- DT2 (average duration 11.5 years) - Average age: 59 years - 40% female - Average BMI: 30 kg/m^2^ (<40 kg/m^2^) - Baseline HbA1c: 8.5% (7–11%)	- DT2 (average duration 10.5 years) - Average age: ≥18 years - HbA1c: 7.0–10.0%	- DT2 (average duration 10.5 years) - Average age: 58 years - 37% female - Average BMI: 29.5 kg/m^2^ (<40 kg/m^2^) - Baseline HbA1c: 8.5% (7–11%) - Layering for S.U./glinide use	- DT2 (average duration 10.5 years) - Average age: 44.9 years - 41% female - Average BMI: 26.5 kg/m^2^ (<40 kg/m^2^) - Baseline HbA1c: 7.51% (7–10%)	- DT2 (average duration 10.5 years) - Average age: ≥18 years - HbA1c: 7.0–10.0%, insulin-naïve	- TD1 (average duration 19.5 years) - Average age: 44.2 years - 42% female - Average BMI: 26.5 kg/m^2^ (<40 kg/m^2^) - Baseline HbA1c: 7.61% (7–11%)
**Results**	- HbA1c reduction: −0.2% (CI Δ 0.4–0.03%), *p* <0.001 - Increase in patients at target (HbA1c < 7%) without significant hypoglycemia: +10% - ΔFPG not significant - Average insulin dose at 52 weeks: 31 IU/day icodec vs. 32 IU/day glargine; on average 0.35 IU/kg/day - Δweight not significant (+2 kg both groups) - TIR increase +4.3% (CI 1.9–6.6%) *p* < 0.001	- At week 26, icodec demonstrated non-inferiority and superiority to degludec in reducing HbA1c from baseline. Clinically significant hypoglycemia rates were not significant between the two groups at week 31	- HbA1c reduction −0.2% (CI Δ 0.3–0.1%), *p* < 0.001 - Increase in patients at target (HbA1c < 7%) without significant hypoglycemia: +15% - ΔFPG not significant - Average insulin dose at 26 weeks: 29 IU/day icodec vs. 27 IU/day degludec; on average 0.3 IU/kg/day - Δweight not significant (+2.5 kg both groups)	At week 26, the mean change in HbA1c was −1.16 percentage points in the icodec group (baseline 8.29%) and −1.18 percentage points in the glargine U100 group (baseline 8.31%). Combined level 2 and level 3 hypoglycemia rates were similar between treatment groups.	At week 52, insulin icodec used in conjunction with the dosing guide app demonstrated non-inferiority and superiority versus the basal insulin analogues in reducing the estimated mean HbA1c from baseline	- HbA1c reduction: −0.47% (*p* < 0.0001) - Increase in patients at target (HbA1c <7%) without significant hypoglycemia: +9.5% - ΔFPG (icodec −15.08 mg/dL—degludec −33.66 mg/dL ETD 18.58 (8.58 to 28.58), *p* = 0.0003 - Estimated mean weekly total insulin dose, U/week (U/day) at 26 weeks icodec 311 (~44) vs. degludec 323 (~46) ETR 0.96 (0.90 to 1.03), *p* = 0.27 - Δweight icodec 1.29 vs. degludec 1.01 ETD 0.28 (−0.37 to 0.92), *p* = 0.41
**Hypoglycemic events**	Icodecs: - 226 episodes in 61 pcs (12.4%) - 1 episode of severe hypoglycemia Glargine: - 114 episodes in 66 pcs (13.4%) - 7 episodes of severe hypoglycemia	Clinically significant hypoglycemia rates were not significant between the two groups at week 31	Icodecs: - 53 episodes in 26 pcs (9%) - 0 episodes of severe hypoglycemia Degludec: - 23 episodes in 17 pieces (6%) - 2 episodes of severe hypoglycemia	Icodecs: - 35 serious adverse events were reported in 22 (8%) of 291 participants Degludec U100: - 33 serious adverse events were reported in 25 (9%) of 291 participants	Clinically significant or severe hypoglycemia rates were not significantly different between the treatment groups at week 57	Icodecs: - 2789 episodes in 246 pcs (19.60%) - 47 episodes of severe hypoglycemia (0.33%) Degludec: - 1478 episodes in 223 pieces (10.26%) - 17 episodes of severe hypoglycemia (0.12%)
**Adverse events**	Mostly mild/moderate events and not associated with treatment Deaths: 5 in icodec, 4 in glargine No reactions at the injection site or critical issues related to medication errors described	Mostly mild/moderate events and not associated with treatment Deaths: 5 in icodec, 4 in glargine No reactions at the injection site or critical issues related to medication errors described	Mostly mild/moderate events and not associated with treatment Deaths: 2 in icodec, 1 in degludec 8.5 vs. 4.4% injection site reactions for icodec vs. degludec Usage errors <5%	Mostly mild/moderate events and not associated with treatment No reactions at the injection site or critical issues related to medication errors described	Mostly mild/moderate events and not associated with treatment No reactions at the injection site or critical issues related to medication errors described	Mostly mild/moderate events and not associated with treatment Deaths: 1 in icodec, 0 in degludec 0.07% vs. 0.06% injection site reactions for icodec vs. degludec
